# Breast Events After Treatment of Ductal Carcinoma In Situ in Women: A Population‐Based Study

**DOI:** 10.1002/cam4.71558

**Published:** 2026-01-29

**Authors:** Qian Chen, Ian Campbell, Mark Elwood, Alana Cavadino, Phyu Sin Aye, Sandar Tin Tin

**Affiliations:** ^1^ Department of Epidemiology and Biostatistics, Faculty of Medical and Health Sciences University of Auckland Auckland New Zealand; ^2^ Department of Surgery, Faculty of Medical and Health Sciences University of Auckland Auckland New Zealand; ^3^ Department of Pharmacology, Faculty of Medical and Health Sciences University of Auckland Auckland New Zealand; ^4^ Cancer Epidemiology Unit, Oxford Population Health University of Oxford Oxford UK

**Keywords:** breast events, DCIS, invasive breast cancer, risk factors, treatment

## Abstract

**Background:**

Despite favourable survival prognosis, the main concern for ductal carcinoma in situ (DCIS) is local recurrence, especially progression to invasive cancer. This study identified factors associated with breast events following DCIS treatment.

**Methods:**

Women with unilateral DCIS between 2000 and 2022 were identified from New Zealand Breast Cancer Foundation National Register. The primary endpoint was cumulative incidence of invasive breast cancer, ipsilateral (iIBC) or contralateral (iCBC). Secondary endpoints included ipsilateral breast event (IBE), in situ or invasive, and any breast event (IBE or CBC). Fine‐Gray models were used to identify the associated factors and estimate subdistribution hazard ratios (sHRs).

**Results:**

Among 5740 patients followed for a median duration of 4.8 years, the 5‐ and 10‐year cumulative risks were 3.0% (95% CI, 2.4%, 3.5%) and 6.6% (95% CI, 5.7%, 7.6%) for iIBC, and 2.7% (95% CI, 2.2%, 3.3%) and 6.3% (95% CI, 5.4%, 7.3%) for iCBC, respectively. A higher risk of iIBC was observed in women aged under 45 at diagnosis (sHR, 1.81; 95% CI: 1.18, 2.79) or had DCIS size > 20 mm (sHR, 1.42; 1.05, 1.93), and a lower risk in those who received additional RT after BCS (HR: 0.61; 0.44, 0.84) or mastectomy (sHR, 0.21; 0.13, 0.32). Similar associations were observed for IBE and any breast event, for which surgical margin < 2 mm was also associated with a higher risk. Having surgery at a private facility, where higher‐risk patients were likely to be treated, was associated with a higher risk of iCBC.

**Conclusions:**

DCIS size, surgical approach, and age at diagnosis influenced the risk of breast events after DCIS, which may be considered in efforts to improve treatment strategies for higher‐risk women.

## 1 Introduction

The incidence of ductal carcinoma in situ (DCIS) has increased since the introduction of population‐based mammographic screening in many countries; it now accounts for around a quarter of all breast cancer cases (invasive and in situ) identified through screening [1]. Standard treatment for DCIS typically involves mastectomy or breast‐conserving surgery (BCS), with or without radiotherapy (RT) and/or endocrine therapy (ET). These treatments aim to reduce future ipsilateral breast event (IBE) and/or contralateral breast cancer (CBC) [2, 3].

Population‐based studies from the United Kingdom showed that, among women with screened or non‐screened DCIS, the invasive breast cancer rates were the lowest among those who received mastectomy, followed by BCS with RT [4, 5]. A pooled analysis of four randomised control trials (RCTs) of women with DCIS showed adjuvant RT after BCS significantly reduced 10‐year IBE compared to BCS alone (12.9% vs. 28.1%), regardless of the detection methods, margin status, DCIS grade, and size, but not CBC or metastasis rates [6]. Many other factors have been associated with subsequent breast events, such as young age, high DCIS grade, the presence of necrosis, and close surgical margins. Based on these factors, several prognostic models have been developed to guide treatment decisions for DCIS [7, 8].

In New Zealand, the biennial national breast screening programme, BreastScreen Aotearoa, was implemented in 1999, initially for women aged 50 to 64 and extended to include those aged 45 to 69 in 2004 [9, 10]. An increased incidence of DCIS was driven by programme screening participation, with variations by ethnicity [11]. A report from the Breast Cancer Foundation National Register (BCFNR) indicated that nearly all women diagnosed with DCIS were treated with surgery, with around 60% receiving BCS, with or without RT, and the remainder receiving mastectomy. The overall 10‐year locoregional recurrence free survival was 95% [12].

The risk of developing DCIS and invasive breast cancer after treatment of DCIS remains poorly understood in women with DCIS in New Zealand. Hence, we conducted a population‐based study to characterise the subsequent breast event risks of women having DCIS treatment in New Zealand.

## 2 Methods

### 2.1 Study Data

This study used the data from BCFNR for the period of 2000–2022. The register started prospectively capturing data from Auckland and Waikato in 2000, expanded to Christchurch in 2009 and to Wellington in 2010, covering approximately two‐thirds of New Zealand's breast cancer cases [12]. Since 2020, the register has expanded to include all breast cancer diagnoses nationwide [13]. This register uses an opt‐out approach and has a withdrawal rate of less than 1% [13]. We included women with unilateral DCIS diagnosed between 2000 and 2020, who received BCS or mastectomy for primary DCIS. Patients with a prior diagnosis of either DCIS or invasive breast cancer before their index diagnosis of unilateral DCIS were excluded (Figure S1).

### 2.2 Variables of Interest

Information on demographics (age at diagnosis, ethnicity, domicile code), clinicopathological characteristics (mode of diagnosis, laterality, tumour grade, tumour size, tumour necrosis, circumferential margins, oestrogen receptor (ER) status), and treatments (types of final surgery, RT, surgical treatment facility, axillary intervention, adjuvant ET) were extracted.

The deprivation level was determined based on the domicile code, using the New Zealand deprivation (NZDep) index, a national deprivation score ranging from 1 (the least deprived areas) to 10 (the most deprived areas) [14]. The deprivation score was grouped into NZDep 1–4, 5–7 and 8–10. The area of residence was categorised as rural, urban and other or unknown, according to Statistics New Zealand definitions based on the domicile code [15]. Mode of diagnosis was grouped into national programme screening, non‐programme image detected and symptomatic. Tumour grade was divided into high, intermediate, low and unknown. DCIS size was categorised into ≤ 20 mm and > 20 mm. Tumour necrosis status was categorised as present, absent and unknown. Circumferential margins were grouped into < 2 mm, ≥ 2 mm and clear but with unknown measurement (no specific measurement recorded). The final surgery included BCS (with or without adjuvant RT) and mastectomy. The axillary intervention included sentinel lymph node biopsy (SLNB), axillary lymph node dissection (ALND), and no axillary surgery. The ER status and adjuvant ET were classified as ER‐positive with ET, ER‐positive without ET, and other (ER‐negative, no ER testing).

### 2.3 Outcomes

The primary endpoint was cumulative incidence of invasive breast cancer, including ipsilateral (iIBC) or contralateral (iCBC), censored by other events. The secondary endpoints included the cumulative incidence of IBE and any breast event. IBE was defined as the first occurrence of DCIS or invasive cancer in the ipsilateral breast, and any breast event was defined as the first occurrence of IBE or CBC. Regional lymph node involvement in the absence of a prior diagnosis of iIBC (*n* = 8) was classified as iIBC. Bilateral breast events (*n* = 16) were included in separate analyses for both IBE and CBC. All endpoints were measured from the date of tissue diagnosis to the date of first breast event, metastatic disease, death, or last follow‐up, whichever came first.

### 2.4 Statistical Analysis

Differences in patients' demographics and treatment characteristics across subgroups were assessed using chi‐square (χ2) tests for categorical variables and the Kruskal–Wallis test for continuous variables. Cumulative incidence of breast events was estimated using the cumulative incidence function, accounting for death before a breast event as a competing risk. Treatment groups were compared using Gray's test [16]. For the factors associated with breast events analyses, the multivariable Fine‐Gray subdistribution hazard model including all demographic and clinical variables was used to estimate hazard ratios (HR) [17]. Subgroup analyses stratified by mode of diagnosis (screened, defined as programme screen/non‐programme image, vs. symptomatic) were conducted for the risks of iIBC and iCBC. All analyses were performed using R 4.3.1 [18] and a two‐sided *p* < 0.05 was considered statistically significant.

## 3 Results

### 3.1 Characteristics of Study Population

A total of 5740 women diagnosed with unilateral DCIS as their first breast cancer were included in the study (Table 1). Most women were diagnosed in the screening age (45–69 years) (83.0%), identified as European (71.3%), residing in the least deprived area (49.3%), or urban/other area (86.3%) and detected through programme screening (60.7%). The most common diagnosis was DCIS size ≤ 20 mm (57.6%), high grade (47.4%), and presentation with necrosis (60.8%). About 38.2% of women received BCS with RT, 34.9% received mastectomy, and 26.9% had only BCS.

**TABLE 1:** Demographic, tumour characteristics of women with DCIS by locoregional treatment group.

Characteristic N (%)	Overall *N* = 5740	BCS alone *N* = 1543	BCS with RT *N* = 2197	Mastectomy (b) *N* = 2000	*p*(a)
Age at diagnosis Median(range)	56 (23,92)	58 (23,92)	57 (28,83)	54 (27,90)	< 0.0001
Age group					< 0.0001
< 45 years	461 (8.0%)	98 (6.3%)	104 (4.7%)	259 (13.0%)	
45–69 years	4766 (83.0%)	1254 (81.3%)	1960 (89.2%)	1552 (77.6%)	
> 69 years	513 (9.0%)	191 (12.4%)	133 (6.1%)	189 (9.4%)	
Ethnicity					0.4487
Māori	470 (8.2%)	119 (7.7%)	199 (9.1%)	152 (7.6%)	
Pacific	261(4.5%)	77 (5.0%)	94 (4.3%)	90 (4.5%)	
Asian	819(14.3%)	241 (15.6%)	299 (13.6%)	279 (14.0%)	
European	4092 (71.3%)	1079 (69.9%)	1569 (71.4%)	1444 (72.2%)	
Other or Unknown	98(1.7%)	27 (1.8%)	36 (1.6%)	35 (1.7%)	
Deprivation					0.2015
1–4 (less deprived)	2830 (49.3%)	767 (49.7%)	1051 (47.8%)	1012 (50.6%)	
5–7	1635(28.5%)	423 (27.4%)	637 (29.0%)	575 (28.8%)	
8–10 (more deprived)	1275(22.2%)	353 (22.9%)	509 (23.2%)	413 (20.6%)	
Rurality					0.5356
Rural	786(13.7%)	217 (14.1%)	309 (14.1%)	260 (13.0%)	
Urban/other	4954 (86.3%)	1326 (85.9%)	1888 (85.9%)	1740 (87.0%)	
Mode of diagnosis					< 0.0001
Programme screen	3484(60.7%)	931 (60.3%)	1505 (68.5%)	1048 (52.4%)	
Non‐programme image	1214 (21.1%)	341 (22.1%)	460 (20.9%)	413 (20.7%)	
Symptomatic	1042 (18.2%)	271 (17.6%)	232 (10.6%)	539 (26.9%)	
Laterality of diagnosis					0.1101
Left	3029 (52.8%)	848 (55.0%)	1132 (51.5%)	1049 (52.5%)	
Right	2711 (47.2%)	695 (45.0%)	1065 (48.5%)	951 (47.5%)	
DCIS size					< 0.0001
≤ 20 mm	3306 (57.6%)	1372 (88.9%)	1452 (66.1%)	482 (24.1%)	
> 20 mm	2434 (42.4%)	171 (11.1%)	745 (33.9%)	1518 (75.9%)	
Grade					< 0.0001
High	2721 (47.4%)	297 (19.2%)	1190 (54.2%)	1234 (61.7%)	
Intermediate	2081(36.3%)	672 (43.6%)	830 (37.8%)	579 (29.0%)	
Low	885 (15.4%)	543 (35.2%)	169 (7.7%)	173 (8.6%)	
Unknown	53 (0.9%)	31 (2.0%)	8 (0.3%)	14 (0.7%)	
Necrosis					< 0.0001
None	1883 (32.8%)	891 (57.8%)	552 (25.1%)	440 (22.0%)	
Present	3492 (60.8%)	542 (35.1%)	1534 (69.8%)	1416 (70.8%)	
Unknown	365 (6.4%)	110 (7.1%)	111 (5.1%)	144 (7.2%)	
Facility of Surgery					0.0146
Private	1741 (30.3%)	464 (30.1%)	663 (30.2%)	614 (30.7%)	
Public	3735 (65.1%)	1000 (64.8%)	1459 (66.4%)	1276 (63.8%)	
Unknown	264 (4.6%)	79 (5.1%)	75 (3.4%)	110 (5.5%)	
Surgical margin					< 0.0001
< 2 mm	750 (13.1%)	200 (13.0%)	356 (16.2%)	194 (9.7%)	
≥ 2 mm	4924 (85.8%)	1318 (85.4%)	1828 (83.2%)	1778 (88.9%)	
Clear, unknown measurement	66 (1.1%)	25 (1.6%)	13 (0.6%)	28 (1.4%)	
Axillary intervention					< 0.0001
No axillary intervention	3621 (63.1%)	1420 (92.0%)	1862 (84.7%)	339 (17.0%)	
ALND	136 (2.4%)	8 (0.5%)	10 (0.5%)	118 (5.9%)	
SLNB	1983 (34.5%)	115 (7.5%)	325 (14.8%)	1543 (77.1%)	
ER status and Adjuvant ET*					< 0.0001
ER+ with ET	184 (3.2%)	26 (1.7%)	109 (5.0%)	49 (2.5%)	
ER+ without ET	540 (9.4%)	147 (9.5%)	240 (10.9%)	153 (7.6%)	
Other	5016 (87.4%)	1370 (88.8%)	1848 (84.1%)	1798 (89.9%)	

*Note:* (a) ruskal‐Wallis rank sum test; Pearson's Chi‐squared test. (b) Mastectomy includes unilateral mastectomy only (*n* = 1857), bilateral mastectomy (*n* = 105), mastectomy with adjuvant RT (*n* = 38).

Abbreviations: ALND: axillary lymph node dissection; BCS: breast conserving surgery; ER+: oestrogen receptor positive; ET: endocrine therapy; RT: radiotherapy; SLNB: sentinel lymph node biopsy.

* Other includes ER‐negative (*n* = 163) and no ER testing (*n* = 4853).

### 3.2 Risk of Invasive Breast Cancer, IBE, and Any Breast Event

The median (IQR) follow‐up was 4.8 (1.7, 9.3) years. 713 women (12.4%) developed any breast event, including 473 (8.3%) who developed invasive breast cancer. 409 women (7.2%) died from non‐breast cancer causes. The 5‐year and 10‐year cumulative risk for iIBC was 3.0% (95% CI, 2.4%, 3.5%), and 6.6% (95% CI, 5.7%, 7.6%), and for iCBC was 2.7% (95% CI, 2.2%, 3.3%), and 6.3% (95% CI, 5.4%, 7.3%). The corresponding risk for invasive breast cancer risk was 5.5% (95% CI, 4.8%, 6.2%), and 12.1% (95% CI, 10.9%, 13.4%), respectively. For IBE, the risks were 5.4% (95% CI, 4.7%, 6.1%), and 10.4% (95% CI, 9.3%, 11.6%), and for any breast event 9.2% (95% CI, 8.4%, 10.2%), and 17.7% (95% CI, 16.3%, 19.1%).

**FIGURE 1:**
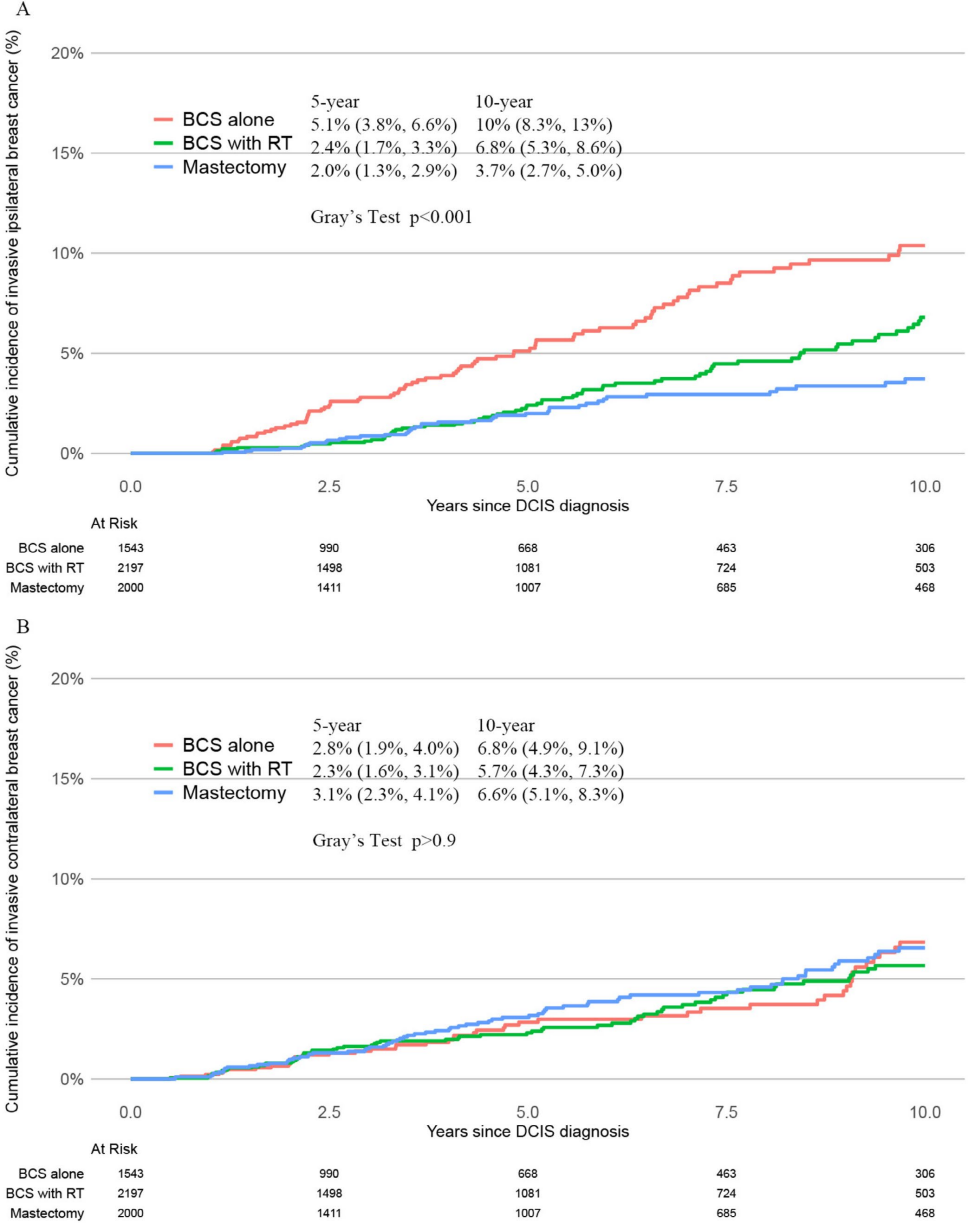
Cumulative Incidence Function Curves for ipsilateral invasive breast cancer (A), invasive contralateral breast cancer (B), by locoregional treatment group. Note: Gray's Test compares the cumulative incidence across treatment groups during the full follow‐up.

Cumulative incidences of iIBC differed by locoregional treatment group, and were lower in women who received additional RT or mastectomy (Figure 1A). In contrast, the risk of iCBC did not differ significantly across the locoregional treatment groups (Figure 1B).

The incidence of total‐IBE, DCIS‐IBE and any breast event followed similar patterns by locoregional treatment group (Figures S2A, B, and C).

### 3.3 Factors Associated With Invasive Breast Cancer

In the multivariable analysis, women who were under 45 years old at diagnosis, had DCIS size > 20 mm had a higher risk of iIBC (Table 2). Receiving BCS with RT or mastectomy was associated with a lower risk compared to BCS alone. In terms of iCBC, women who had surgery in a private facility had a higher risk; the results remained unchanged after excluding women who had bilateral mastectomy. Women treated in private facilities were more often diagnosed at a younger age and outside the national screening programme, and had larger DCIS size compared to those treated in public facilities Table S1.

**TABLE 2:** The multivariable Fine and Gray proportional subdistribution hazards analysis of association of factors with invasive ipsilateral and contralateral breast cancer in women with DCIS.

Characteristic	Categories	iIBC		iCBC	
		Events	sHR (95% CI)	Events	sHR (95% CI)
Age group	45–69 years	191	Reference	211	Reference
	< 45 years	31	**1.81 (1.18, 2.79)**	23	1.21 (0.76, 1.92)
	> 69 years	21	0.91 (0.56, 1.48)	11	0.53 (0.28, 1.02)
Ethnicity	European	186	Reference	175	Reference
	Māori	20	1.11 (0.68, 1.79)	17	1.03 (0.61, 1.72)
	Pacific	12	1.07 (0.57, 2.00)	12	1.20 (0.64, 2.24)
	Asian	24	0.72 (0.47, 1.10)	36	1.14 (0.80, 1.64)
	Other or Unknown	< 5	0.26 (0.04, 1.77)	5	1.44 (0.61, 3.39)
Deprivation	1–4 (less deprived)	117	Reference	123	Reference
	5–7	69	1.06 (0.78, 1.45)	67	0.92 (0.68, 1.24)
	8–10 (more deprived)	57	1.09 (0.76, 1.56)	55	0.92 (0.65, 1.31)
Rurality	Urban/other	218	Reference	216	Reference
	Rural	25	0.81 (0.54, 1.21)	29	0.91 (0.61, 1.36)
Mode of diagnosis	Programme screen	112	Reference	129	Reference
	non‐programme image	79	0.96 (0.68, 1.34)	73	0.77 (0.54, 1.08)
	Symptomatic	52	1.16 (0.78, 1.72)	43	0.76 (0.51, 1.15)
DCIS size	≤ 20 mm	155	Reference	137	Reference
	> 20 mm	88	**1.42 (1.05, 1.93)**	108	1.34 (0.97, 1.84)
Grade	High	95	Reference	105	Reference
	Intermediate	97	1.08 (0.78, 1.48)	78	0.91 (0.65, 1.26)
	Low	49	0.85 (0.53, 1.35)	59	1.30 (0.80, 2.11)
	Unknown	< 5	0.47 (0.11, 2.06)	< 5	1.10 (0.33, 3.63)
Necrosis	Present	131	Reference	135	Reference
	None	90	1.09 (0.77, 1.54)	91	1.09 (0.75, 1.58)
	Unknown	22	0.84 (0.52, 1.37)	19	0.78 (0.47, 1.30)
Locoregional treatment	BCS alone	98	Reference	64	Reference
	BCS with RT	98	**0.61 (0.44, 0.84)**	90	0.96 (0.67, 1.39)
	Mastectomy	47	**0.21 (0.13, 0.32)**	91	0.89 (0.58, 1.37)
Facility of Surgery	Public	142	Reference	147	Reference
	Private	89	1.24 (0.93, 1.67)	93	**1.40 (1.04, 1.88)**
	Unknown	12	0.91 (0.48, 1.73)	5	0.47 (0.19, 1.14)
Surgical margin	≥ 2 mm	199	Reference	209	Reference
	< 2 mm	38	1.30 (0.91, 1.85)	33	1.13 (0.77, 1.65)
	Clear, unknown measurement	6	1.16 (0.47, 2.85)	< 5	0.60 (0.19, 1.91)
Axillary intervention	No axillary intervention	173	Reference	155	Reference
	ALND	< 5	0.68 (0.24, 1.91)	11	1.07 (0.56, 2.03)
	SLNB	66	**1.48 (1.03, 2.12)**	79	1.09 (0.75, 1.59)
ER status and adjuvant ET	ER+ without ET	27	Reference	29	Reference
	ER+ with ET	< 5	0.13 (0.02, 1.02)	6	0.80 (0.33, 1.92)
	Other	215	0.95 (0.62, 1.43)	210	0.83 (0.56, 1.23)

Abbreviations: ALND: axillary lymph node dissection; BCS: breast‐conserving surgery; ER+: oestrogen receptor positive; ET: endocrine therapy; iIBC: ipsilateral invasive breast cancer; iCBC: contralateral invasive breast cancer; RT: radiotherapy; sHR: subdistribution hazard ratio; SLNB: sentinel lymph node biopsy.

Subgroup analyses stratified by mode of diagnosis (screened vs. symptomatic) showed largely consistent risk associations for iIBC, except that DCIS size > 20 mm was associated with a higher risk only among screened patients (Table S2). For iCBC, a similar association with DCIS size > 20 mm was observed in the screened group, whereas Asian/other ethnicity was associated with a higher risk in the symptomatic group (Supplementary Table 3).

### 3.4 Factors Associated With IBE, and Any Breast Event

In the multivariable analysis, women who were under 45 years old at diagnosis, had DCIS size ≥ 20 mm, had surgery in a private facility, and had surgical margin size < 2 mm had a higher risk of IBE (Table 3). Women who received BCS with RT or mastectomy had a lower risk. Similar associations were observed for DCIS‐IBE, for which unknown necrosis status was also associated with a lower risk (Table 3).

**TABLE 3:** The multivariable Fine and Gray proportional subdistribution hazards analysis of association of factors with IBE, DCIS‐IBE in women with DCIS.

Characteristic	Categories	IBE		DCIS‐IBE	
		Events	sHR (95% CI)	Events	sHR (95% CI)
Age group	45–69 years	312	Reference	121	Reference
	< 45 years	50	**1.87 (1.31, 2.67)**	19	**1.96 (1.05, 3.66)**
	> 69 years	29	0.81 (0.53, 1.22)	8	0.63 (0.29, 1.39)
Ethnicity	European	283	Reference	97	Reference
	Māori	36	1.31 (0.91, 1.86)	16	1.70 (0.99, 2.92)
	Pacific	22	1.30 (0.82, 2.07)	10	1.72 (0.85, 3.49)
	Asian	48	0.93 (0.69, 1.27)	24	1.29 (0.82, 2.05)
	Other or Unknown	< 5	0.35 (0.09, 1.35)	< 5	0.52 (0.07, 3.79)
Deprivation	1–4 (less deprived)	192	Reference	75	Reference
	5–7	113	1.07 (0.84, 1.36)	44	1.07 (0.72, 1.58)
	8–10 (more deprived)	86	0.96 (0.72, 1.27)	29	0.77 (0.49, 1.21)
Rurality	Urban/other	346	Reference	128	Reference
	Rural	45	0.90 (0.66, 1.24)	20	1.05 (0.64, 1.74)
Mode of diagnosis	Programme screen	196	Reference	84	Reference
	non‐programme image	112	0.86 (0.66, 1.12)	33	0.71 (0.45, 1.11)
	Symptomatic	83	1.12 (0.82, 1.54)	31	1.08 (0.63, 1.83)
DCIS size	≤ 20 mm	254	Reference	99	Reference
	> 20 mm	137	**1.48 (1.15, 1.90)**	49	**1.59 (1.03, 2.46)**
Grade	High	155	Reference	60	Reference
	Intermediate	154	1.01 (0.79, 1.29)	57	0.92 (0.62, 1.36)
	Low	78	0.80 (0.56, 1.16)	29	0.76 (0.42, 1.36)
	Unknown	< 5	0.64 (0.22, 1.84)	< 5	1.04 (0.23, 4.62)
Necrosis	Present	223	Reference	92	Reference
	None	139	0.93 (0.71, 1.22)	49	0.72 (0.47, 1.11)
	Unknown	29	**0.66 (0.44, 0.99)**	7	**0.39 (0.18, 0.85)**
Locoregional treatment	BCS alone	169	Reference	71	Reference
	BCS with RT	161	**0.55 (0.43, 0.71)**	63	**0.46 (0.31, 0.69)**
	Mastectomy	61	**0.16 (0.11, 0.24)**	14	**0.09 (0.04, 0.21)**
Facility of Surgery	Public	228	Reference	86	Reference
	Private	142	**1.31 (1.04, 1.65)**	53	1.44 (1.00, 2.07)
	Unknown	21	0.92 (0.57, 1.50)	9	0.09 (0.04, 0.21)
Surgical margin	≥ 2 mm	312	Reference	113	Reference
	< 2 mm	70	**1.47 (1.12, 1.93)**	32	**1.74 (1.15, 2.65)**
	Clear, unknown measurement	9	1.26 (0.61, 2.60)	< 5	1.44 (0.44, 4.77)
Axillary intervention	No axillary intervention	296	Reference	123	Reference
	ALND	9	1.08 (0.50, 2.30)	5	2.08 (0.68, 6.41)
	SLNB	86	1.11 (0.82, 1.50)	20	0.63 (0.35, 1.14)
ER status and adjuvant ET	ER+ without ET	44	Reference	17	Reference
	ER+ with ET	5	0.41 (0.16, 1.06)	< 5	0.85 (0.27, 2.63)
	Other	342	0.92 (0.66, 1.27)	127	0.87 (0.51, 1.49)

Abbreviations: ALND: axillary lymph node dissection; BCS: breast‐conserving surgery; CBC: contralateral breast cancer; ER+: oestrogen receptor positive; ET: endocrine therapy; sHR: subdistribution hazard ratio; IBE: ipsilateral breast event; RT: radiotherapy; SLNB: sentinel lymph node biopsy.

Among patients treated with BCS+/−RT (*n* = 3740), the median (IQR) follow‐up was 4.6 (1.5, 9.2) years. In multivariable analysis, age younger than 45 years, Māori ethnicity, surgery in a private facility, and DCIS size ≥ 20 mm were associated with a higher risk of IBE, whereas receipt of RT after BCS was associated with a lower risk (Table S4). Similar associations were observed for iIBC risk (Table S4).

For any breast event, a higher risk was associated with younger age, DCIS size > 20 mm, had surgery in a private facility, and surgical margin size < 2 mm (Table S5). Lower risk was observed among women who had RT after BCS or mastectomy, and in those ER‐positive DCIS treated with ET.

## 4 Discussion

In this population‐based cohort study, women who were under 45 years old at diagnosis, had DCIS size > 20 mm, had surgery in a private facility, and had BCS alone had a higher risk of iIBC. Similar factors were associated with higher risk of IBE and any breast cancer event, for which surgical margin < 2 mm was also associated with a higher risk. A higher iCBC risk was associated with having surgery in a private facility but not with locoregional treatments as expected.

We found that the 5 and 10‐year cumulative incidence of invasive breast cancer was 5.5% and 12.1%, respectively. Similar 5‐year rates have been reported in the United Kingdom (UK) population‐based studies, though their 10‐year rates were slightly lower [4, 5]. A pooled analysis of studies from Europe and the United States (US), involving 18% of patients who underwent BCS alone, reported a lower 10‐year iIBC of 3.2% [19]. Notably, while more intensive locoregional treatment was associated with a lower risk of iIBC, the 10‐year risk of iCBC was similar across treatment groups in our study and remained higher than estimates reported in the population‐based studies from the US [20], UK [4, 5] and the Netherlands [21].

The 5‐ and 10‐year rates of IBE in our cohort were comparable to those reported in previous studies from developed countries focusing on BCS, with or without RT, with 5‐year rates ranging from 4.5% to 9% and 10‐year rates from 7.4% to 15% [8, 22, 23, 24]. RCTs have shown that adding RT to BCS significantly reduces the risk of IBE and any breast event by 44% and 40%, respectively, without significantly affecting the risk of CBC [6]. A meta‐analysis also reported significant differences in the pooled 10‐year rates of IBE among locoregional treatment groups, with rates of 3.0% for mastectomy, 13.0% for BCS with RT, and 25.2% for BCS alone [25]. Although mastectomy did not demonstrate a survival benefit over BCS among women with DCIS [26], it leads to the lowest iIBC, IBE, and any breast cancer event risk [19, 27].

Among women who were treated with BCS, with or without RT, meta‐analyses revealed that margins ≥ 2 mm were associated with a 50% reduction of local recurrence compared to those with a margin < 2 mm [28]. Consistent with this, New Zealand treatment guidelines recommend aiming for a circumferential margin of ≥ 2 mm where possible [2]. In our study, a 32% lower IBE rate was observed in those with margins of ≥ 2 mm. Similar to previous studies demonstrating the limited prognostic effect of DCIS grade and necrosis status after treatment [4, 6, 19, 29, 30], our multivariable analysis did not identify a significant association of these factors with the risk of any events. The lower IBE risk in DCIS with unknown necrosis status may reflect smaller lesions where necrosis was not reported.

Use of ET among women with ER‐positive DCIS remained low at 25%; yet this subgroup tended to have approximately half the risk of any breast events, aligning with findings from RCTs and real‐world studies [31].

Larger DCIS size has been associated with a higher risk of iIBC and IBE [8, 32, 33], with evidence suggesting that this is primarily driven by an increased rate of DCIS‐IBE [19]. Due to the higher sensitivity of mammography for identification of smaller tumours, screen‐detected DCIS tends to be of smaller size than that detected through other methods [34, 35]. In our analysis, DCIS size > 20 mm was associated with a higher risk of iIBC only in the screened group. We did not, however, observe significant associations with mode of diagnosis, unlike previous studies [5, 36].

Women diagnosed with DCIS at a younger age often present with more aggressive disease characteristics [37], such as multicentric disease [38], family history [38], and symptomatic detection [39]. In our study, although women younger than 45 years were more likely to be treated with mastectomy, they remained associated with a higher risk of IBE and any breast cancer event after multivariable adjustment. The risk of iCBC did not differ significantly across age groups, aligning with prior findings [20].

In contrast to previous US research that reported a correlation between deprived neighbourhoods and a higher risk of IBE [40], we found no association between recurrence and either deprivation level or area of residence (rural vs. urban). This may be due to differences in the healthcare system between the two countries, for example, ER testing and use of ET was much higher in the US cohort, particularly in the more affluent areas. While patients who had surgery at the private facility have been shown to have better survival outcomes in early‐stage breast cancer in New Zealand [41], our study found elevated risk of iCBC and any breast cancer event among those treated privately. This may be partly explained by the greater likelihood that these patients were diagnosed outside the national screening programme [41], and therefore had larger DCIS size, as well as influenced by other risk factors not available for this analysis, such as a family history of breast cancer, smoking, alcohol consumption, and obesity [42, 43, 44]. More intensive follow‐ups in private facilities may also contribute, which may be confirmed in future studies.

We did not observe ethnic differences in most outcomes, except for a higher risk of IBE among Māori women treated with BCS, with or without RT. Pacific women also showed a trend towards higher IBE risk. In New Zealand, despite Māori and Pacific women having higher proportions of ER‐positive breast cancer, their increased breast cancer mortality has been linked to multiple factors, including late stage at diagnosis, deprivation, treatment access, locoregional treatment patterns [45], and lower adherence to adjuvant endocrine therapy [46]. In our DCIS cohort, there was no significant difference in locoregional treatment across ethnicities.

To our knowledge, this is the first study to examine demographic and clinical factors associated with subsequent breast events among women with DCIS in New Zealand, providing evidence that also includes the underrepresented Māori and Pacific populations. Yet, the sample size is limited for some analyses. We also do not have information on some prognostic factors for DCIS, such as family history [8], BReast CAncer gene 1 (BRCA1) and BRCA2 mutation [47], HER2 status [48], and lifestyle‐related variables (e.g., alcohol intake, body mass index) [49] for the majority of patients. Additionally, the very low ER testing rate in our study population may have limited patients' treatment options regarding the ET eligibility.

This cohort study has identified the important risk factors for breast events following a DCIS diagnosis in New Zealand including larger DCIS size, breast conservation, especially without RT, surgical margin < 2 mm, and younger age at diagnosis. The observed association between private facility surgery and higher iCBC risk may likely reflect higher underlying risk among patients treated at these facilities. These findings may inform personalised treatment strategies for women at elevated risk, and improve equitable outcomes.

## Author Contributions

Q.C. and S.T.T. conceptualised the research. Q.C. undertook the background literature review, formal data analyses, and wrote the original manuscript and generated the tables and figures. All authors interpreted the data and reviewed the final manuscript.

## Funding

This study was supported by Auckland Medical Research Foundation (Ref: 1124002) and University of Auckland Research Development Fund (Ref: 3729227). The funding sources had no direct involvement in this study or the decision to submit the paper for publication.

## Ethics Statement

This study was approved by the Auckland Health Research Ethics Committee (Ref. AH26746). The Te Rēhita Mate Ūtaetae, New Zealand Breast Cancer Registry, maintains its own governance and New Zealand Health and Disability Ethics Committee approval, using opt‐out consent [12].

## Conflicts of Interest

The authors declare no conflicts of interest.

## Supporting information


**Figure S1:** Flow chart of cases selection
**Figure S2:** Cumulative Incidence Function Curves for ipsilateral breast event (A), DCIS ipsilateral breast event (B), and any breast event (C) by locoregional treatment group.
**Table S1:** Demographic, tumour characteristics of women with DCIS, by the facility of surgery.
**Table S2:** The multivariable Fine and Gray proportional subdistribution hazards analysis of association of factors with invasive ipsilateral breast cancer in women with DCIS, by mode of diagnosis.
**Table S3:** The multivariable Fine and Gray proportional subdistribution hazards analysis of association of factors with invasive contralateral breast cancer in women with DCIS, by mode of diagnosis.
**Table S4:** The multivariable Fine and Gray proportional subdistribution hazards analysis of association of factors with IBE and iIBC in women with DCIS treated with BCS+/−RT.
**Table S5:** The multivariable Fine and Gray proportional subdistribution hazards analysis of association of factors with any breast events in women with DCIS.

## Data Availability

The datasets used in this study contain personal information and are not publicly available, but may be requested from the Breast Cancer Foundation National Register NZ.
