# Supplementary material for: Breast Events After Treatment of Ductal Carcinoma In Situ in Women: A Population‐Based Study
Source: Cancer Med. 2026 Jan 29;15(2):e71558. doi: 10.1002/cam4.71558 (PMC12853141; doi:10.1002/cam4.71558)
Supplement: Supplementary file 1 — Figure S1: Flow chart of cases selection Figure S2: Cumulative Incidence Function Curves for ipsilateral breast event (A), DCIS ipsilateral breast event (B), and any breast event (C) by locoregional treatment group. Table S1: Demographic, tumour characteristics of women with DCIS, by the facility of surgery. Table S2: The multivariable Fine and Gray proportional subdistribution hazards analysis of association of factors with invasive ipsilateral breast cancer in women with DCIS, by mode of diagnosis. Table S3: The multivariable Fine and Gray proportional subdistribution hazards analysis of association of factors with invasive contralateral breast cancer in women with DCIS, by mode of diagnosis. Table S4: The multivariable Fine and Gray proportional subdistribution hazards analysis of association of factors with IBE and iIBC in women with DCIS treated with BCS+/−RT. Table S5: The multivariable Fine and Gray proportional subdistribution hazards analysis of association of factors with any breast events in women with DCIS. [file CAM4-15-e71558-s001.docx]

**Supplementary file**


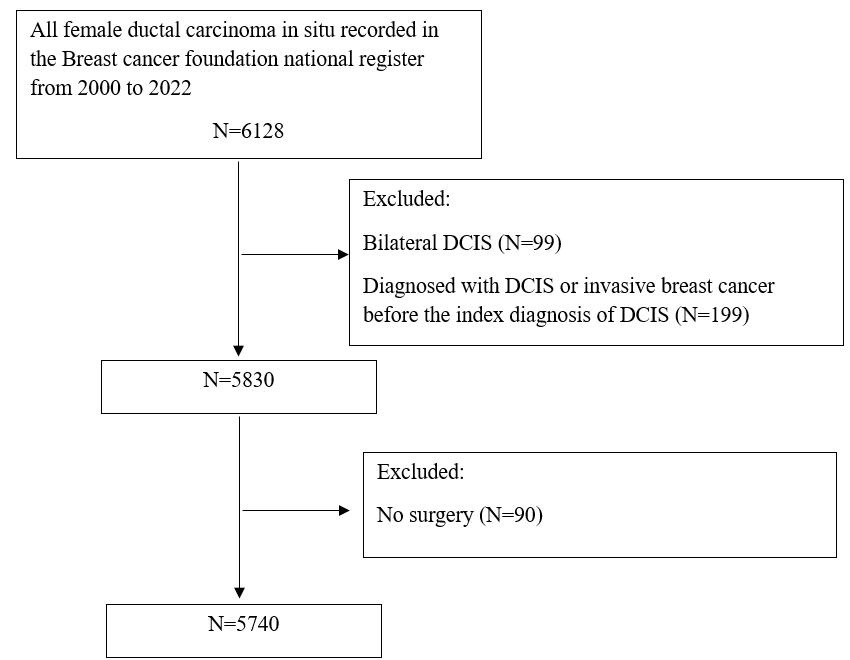


Supplementary Figure 1. Flow chart of cases selection


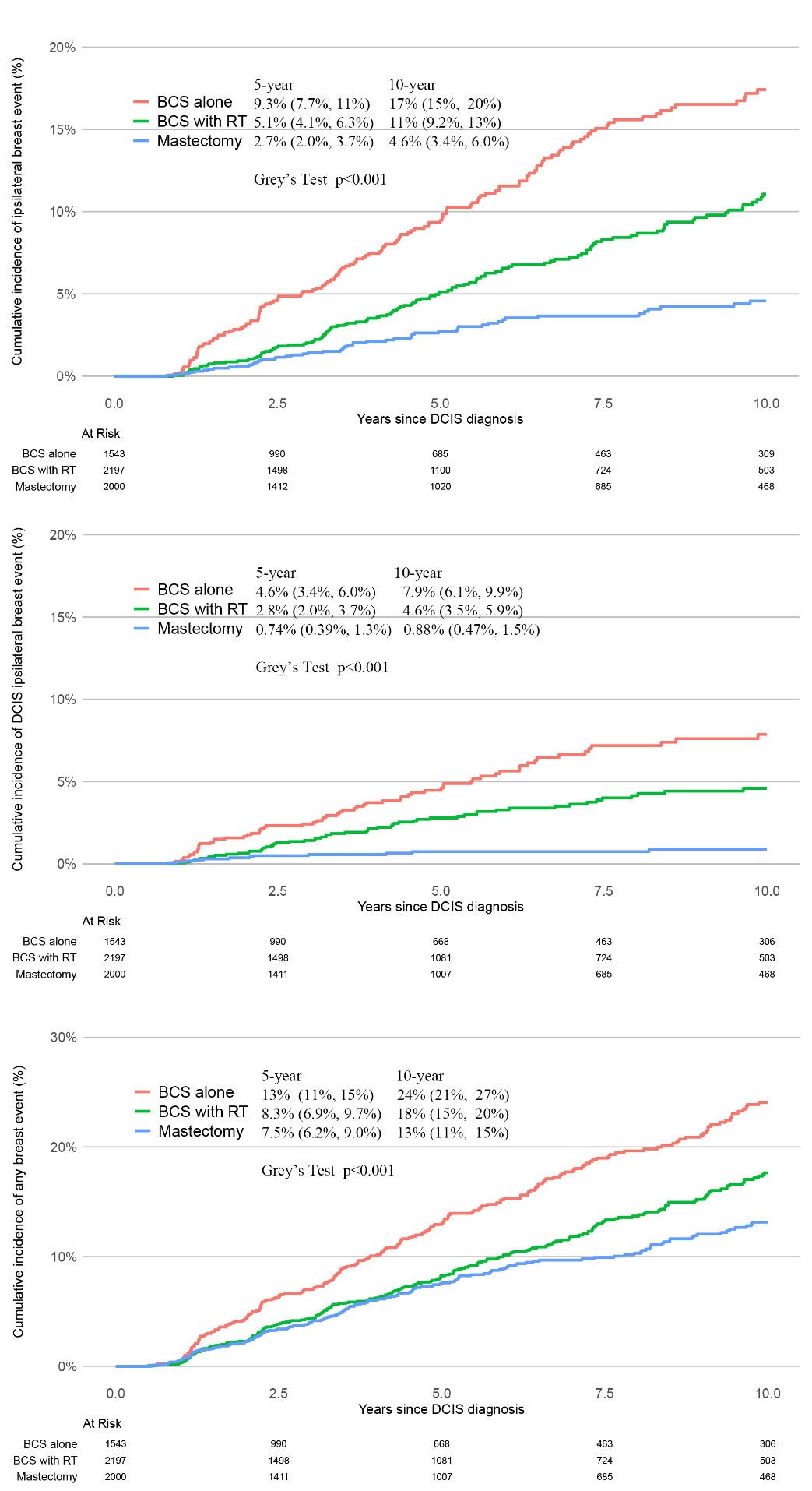


Supplementary Figure 2. Cumulative Incidence Function Curves for ipsilateral breast event (A), DCIS ipsilateral breast event (B), and any breast event (C) by locoregional treatment group.

Note: Gray’s Test compares the cumulative incidence across treatment groups during the full follow-up.

Supplementary Table 1. Demographic, tumour characteristics of women with DCIS, by the facility of surgery.

| **Characteristic**  **N (%)** | **Overall ^b^** N = 5,476 | **Private** N = 1,741 | **Public** N = 3,735 | **p-value** *^a^* |
| --- | --- | --- | --- | --- |
| **Age group** |  |  |  | <0.0001 |
| <45 years | 426 (7.8%) | 180 (10%) | 246 (6.6%) |  |
| 45-69 years | 4,566 (83%) | 1,429 (82%) | 3,137 (84%) |  |
| >69 years | 484 (8.8%) | 132 (7.6%) | 352 (9.4%) |  |
| **Ethnicity** |  |  |  | <0.0001 |
| Māori | 445 (8.1%) | 55 (3.2%) | 390 (10%) |  |
| Pacific | 248 (4.5%) | 21 (1.2%) | 227 (6.1%) |  |
| Asian | 795 (15%) | 235 (13%) | 560 (15%) |  |
| European | 3,891 (71%) | 1,401 (80%) | 2,490 (67%) |  |
| Other or Unknown | 97 (1.8%) | 29 (1.7%) | 68 (1.8%) |  |
| **Deprivation** |  |  |  | <0.0001 |
| 1-4 (less deprived) | 2,602 (48%) | 1,056 (61%) | 1,546 (41%) |  |
| 5-7 | 1,614 (29%) | 469 (27%) | 1,145 (31%) |  |
| 8-10 (more deprived) | 1,260 (23%) | 216 (12%) | 1,044 (28%) |  |
| **Rurality** |  |  |  | 0.1243 |
| Rural | 780 (14%) | 267 (15%) | 513 (14%) |  |
| Urban/other | 4,696 (86%) | 1,474 (85%) | 3,222 (86%) |  |
| **Mode of diagnosis** |  |  |  | <0.0001 |
| Programme screen | 3,320 (61%) | 802 (46%) | 2,518 (67%) |  |
| Non-programme image | 1,174 (21%) | 615 (35%) | 559 (15%) |  |
| Symptomatic | 982 (18%) | 324 (19%) | 658 (18%) |  |
| **DCIS size** |  |  |  | 0.0057 |
| ≤20mm | 3,156 (58%) | 1,051 (60%) | 2,105 (56%) |  |
| >20mm | 2,320 (42%) | 690 (40%) | 1,630 (44%) |  |
| **Grade** |  |  |  | 0.3311 |
| High | 2,580 (47%) | 809 (46%) | 1,771 (47%) |  |
| Intermediate | 1,993 (36%) | 626 (36%) | 1,367 (37%) |  |
| Low | 851 (16%) | 292 (17%) | 559 (15%) |  |
| Unknown | 52 (0.9%) | 14 (0.8%) | 38 (1.0%) |  |
| **Necrosis** |  |  |  | 0.2346 |
| None | 1,842 (34%) | 596 (34%) | 1,246 (33%) |  |
| Present | 3,270 (60%) | 1,017 (58%) | 2,253 (60%) |  |
| Unknown | 364 (6.6%) | 128 (7.4%) | 236 (6.3%) |  |
| **Locoregional treatment** |  |  |  | 0.6973 |
| BCS alone | 1,464 (27%) | 464 (27%) | 1,000 (27%) |  |
| BCS with RT | 2,122 (39%) | 663 (38%) | 1,459 (39%) |  |
| Mastectomy | 1,890 (35%) | 614 (35%) | 1,276 (34%) |  |
| **Surgical margin** |  |  |  | <0.0001 |
| <2mm | 720 (13%) | 251 (14%) | 469 (13%) |  |
| ≥2mm | 4,691 (86%) | 1,447 (83%) | 3,244 (87%) |  |
| Clear, unknown measurement | 65 (1.2%) | 43 (2.5%) | 22 (0.6%) |  |
| **Axillary intervention** |  |  |  | 0.4655 |
| No axillary intervention | 3,480 (64%) | 1,097 (63%) | 2,383 (64%) |  |
| ALND | 129 (2.4%) | 36 (2.1%) | 93 (2.5%) |  |
| SLNB | 1,867 (34%) | 608 (35%) | 1,259 (34%) |  |
| **ER status and Adjuvant ET** |  |  |  | <0.0001 |
| ER+ with ET | 182 (3.3%) | 66 (3.8%) | 116 (3.1%) |  |
| ER+ without ET | 531 (9.7%) | 118 (6.8%) | 413 (11%) |  |
| Other | 4,763 (87%) | 1,557 (89%) | 3,206 (86%) |  |

Abbreviation: ALND: axillary lymph node dissection; BCS: breast conserving surgery; ER+: oestrogen receptor positive; ET: endocrine therapy; RT: radiotherapy; SLNB: sentinel lymph node biopsy.

1. Pearson's Chi-squared test.
2. Unknown facility of surgery (n=264) was excluded.

Supplementary Table 2. The multivariable Fine and Gray proportional subdistribution hazards analysis of association of factors with invasive ipsilateral breast cancer in women with DCIS, by mode of diagnosis.

| **Characteristic** | **Categories** | **Screened** | | **Symptomatic** | |
| --- | --- | --- | --- | --- | --- |
|  |  | **Events** | **sHR (95% CI)** | **Events** | **sHR (95% CI)** |
| Age group | 45-69 years | 168 | Reference | 23 | Reference |
|  | <45 years | 15 | **1.96 (1.13, 3.42)** | 16 | 1.90 (0.97, 3.71) |
|  | >69 years | 8 | 0.70 (0.33, 1.46) | 13 | 0.95 (0.45, 2.03) |
| Ethnicity | European | 152 | Reference | 34 | Reference |
|  | Māori | 16 | 1.14 (0.67, 1.96) | <5 | 1.05 (0.33, 3.33) |
|  | Pacific | 8 | 0.93 (0.42, 1.99) | <5 | 1.36 (0.49, 3.76) |
|  | Asian | 15 | 0.61 (0.46, 1.41) | 9 | 1.22 (0.59, 2.52) |
|  | Other or Unknown | 0 | - | <5 | 1.22 (0.22, 6.65) |
| Deprivation | 1-4 (less deprived) | 95 | Reference | 22 | Reference |
|  | 5-7 | 54 | 1.04 (0.73, 1.49) | 15 | 1.10 (0.55, 2.19) |
|  | 8-10 (more deprived) | 42 | 1.01 (0.67, 1.54) | 15 | 1.38 (0.66, 2.90) |
| Rurality | Urban/other | 169 | Reference | 49 | Reference |
|  | Rural | 22 | 0.88 (0.57, 1.35) | <5 | 0.53 (0.16, 1.83) |
| DCIS size | ≤20mm | 124 | Reference | 31 | Reference |
|  | >20mm | 67 | **1.45 (1.04, 2.01)** | 21 | 1.04 (0.51, 2.09) |
| Grade | High | 79 | Reference | 16 | Reference |
|  | Intermediate | 73 | 0.95 (0.68, 1.34) | 24 | 1.60 (0.71, 3.62) |
|  | Low | 37 | 0.91 (0.53, 1.55) | 12 | 0.84 (0.32, 2.15) |
|  | Unknown | <5 | 0.88 (0.19, 4.15) | 0 | - |
| Necrosis | Present | 112 | Reference | 19 | Reference |
|  | None | 62 | 0.94 (0.63, 1.41) | 28 | 1.55 (0.70, 3.44) |
|  | Unknown | 17 | 0.86 (0.50, 1.47) | 5 | 0.64 (0.20, 2.06) |
| Locoregional treatment | BCS alone | 70 | Reference | 28 | Reference |
|  | BCS with RT | 87 | **0.67 (0.47, 0.97)** | 11 | **0.42 (0.19, 0.92)** |
|  | Mastectomy | 34 | **0.28 (0.17, 0.46)** | 13 | **0.09 (0.03, 0.25)** |
| Facility of Surgery | Public | 110 | Reference | 32 | Reference |
|  | Private | 71 | 1.31 (0.96, 1.80) | 18 | 0.96 (0.53, 1.74) |
|  | Unknown | 10 | 1.12 (0.56, 2.24) | <5 | 0.50 (0.10, 2.39) |
| Surgical margin | ≥2mm | 161 | Reference | 38 | Reference |
|  | <2mm | 26 | 1.26 (0.83, 1.92) | 12 | 1.41 (0.69, 2.85) |
|  | Clear, unknown measurement | <5 | 0.93 (0.31, 2.74) | <5 | 2.08 (0.50, 8.65) |
| Axillary intervention | No axillary intervention | 143 | Reference | 30 | Reference |
|  | ALND | <5 | 0.51 (0.12, 2.09) | <5 | 1.90 (0.30, 11.9) |
|  | SLNB | 46 | 1.51 (0.77, 1.72) | 20 | **4.02 (1.72, 9.42)** |
| ER status and adjuvant ET | ER+ without ET | 20 | Reference | 7 | Reference |
|  | ER+ with ET | <5 | 0.20 (0.03, 1.58) | 0 | - |
|  | Other | 170 | 0.93 (0.58, 1.50) | 45 | 1.46 (0.58, 3.70) |

Abbreviation: ALND: axillary lymph node dissection; BCS: breast conserving surgery; ER+ oestrogen receptor positive; ET: endocrine therapy; sHR: subdistributional Hazard Ratio; iIBC: invasive ipsilateral breast cancer; iCBC: invasive contralateral breast cancer; RT: radiotherapy; SLNB: sentinel lymph node biopsy.

Supplementary Table 3. The multivariable Fine and Gray proportional subdistribution hazards analysis of association of factors with invasive contralateral breast cancer in women with DCIS, by mode of diagnosis.

| **Characteristic** | **Categories** | **Screened** | | **Symptomatic** | |
| --- | --- | --- | --- | --- | --- |
|  |  | **Events** | **sHR (95% CI)** | **Events** | **sHR (95% CI)** |
| Age group | 45-69 years | 188 | Reference | 23 | Reference |
|  | <45 years | 8 | 0.76 (0.36, 1.59) | 15 | 1.44 (0.75, 2.78) |
|  | >69 years | 6 | 0.51 (0.22, 1.18) | 5 | 0.65 (0.24, 1.74) |
| Ethnicity | European | 156 | Reference | 19 | Reference |
|  | Māori | 13 | 0.91 (0.51, 1.63) | <5 | 2.53 (0.73, 8.78) |
|  | Pacific | 11 | 1.36 (0.71, 2.60) | <5 | 0.71 (0.08, 6.34) |
|  | Asian | 20 | 0.77 (0.48, 1.23) | 16 | **3.94 (1.97, 7.86)** |
|  | Other or Unknown | <5 | 0.72 (0.18, 2.84) | <5 | **5.93 (2.00, 17.6)** |
| Deprivation | 1-4 (less deprived) | 104 | Reference | 19 | Reference |
|  | 5-7 | 55 | 0.89 (0.63, 1.24) | 12 | 1.03 (0.49, 2.19) |
|  | 8-10 (more deprived) | 43 | 0.81 (0.55, 1.21) | 12 | 1.59 (0.72, 3.54) |
| Rurality | Urban/other | 178 | Reference | 38 | Reference |
|  | Rural | 24 | 0.86 (0.55, 1.34) | 5 | 1.33 (0.50, 3.53) |
| DCIS size | ≤20mm | 118 | Reference | 19 | Reference |
|  | >20mm | 84 | **1.44 (1.01, 2.05)** | 24 | 0.77 (0.35, 1.73) |
| Grade | High | 80 | Reference | 25 | Reference |
|  | Intermediate | 68 | 0.97 (0.67, 1.40) | 10 | 0.65 (0.30, 1.42) |
|  | Low | 52 | 1.36 (0.81, 2.29) | 7 | 0.95 (0.24, 3.82) |
|  | Unknown | <5 | 1.16 (0.28, 4.92) | <5 | 0.90 (0.08, 10.1) |
| Necrosis | Present | 108 | Reference | 27 | Reference |
|  | None | 80 | 1.24 (0.83, 1.83) | 11 | 0.60 (0.19, 1.89) |
|  | Unknown | 14 | 0.76 (0.42, 1.38) | 5 | 0.80 (0.31, 2.08) |
| Locoregional treatment | BCS alone | 57 | Reference | 7 | Reference |
|  | BCS with RT | 79 | 0.90 (0.61, 1.33) | 11 | 1.34 (0.44, 4.13) |
|  | Mastectomy | 66 | 0.84 (0.52 (1.35) | 25 | 1.01 (0.34, 3.00) |
| Facility of Surgery | Public | 125 | Reference | 22 | Reference |
|  | Private | 73 | 1.23 (0.89, 1.69) | 20 | 1.74 (0.96, 3.16) |
|  | Unknown | 4 | 0.47 (0.17, 1.28) | <5 | 0.43 (0.05, 3.69) |
| Surgical margin | ≥2mm | 171 | Reference | 38 | Reference |
|  | <2mm | 28 | 1.28 (0.84, 1.95) | 5 | 0.58 (0.22, 1.52) |
|  | Clear, unknown measurement | <5 | 0.85 (0.26, 2.78 | 0 | - |
| Axillary intervention | No axillary intervention | 134 | Reference | 21 | Reference |
|  | ALND | 7 | 1.07 (0.49, 2.34) | 4 | 1.16 (0.38, 3.54) |
|  | SLNB | 61 | 1.14 (0.75, 1.74) | 18 | 1.06 (0.44, 2.56) |
| ER status and adjuvant ET | ER+ without ET | 25 | Reference | 4 | Reference |
|  | ER+ with ET | 5 | 0.89 (0.34, 2.30) | 1 | 0.82 (0.11, 6.13) |
|  | Other | 172 | 0.72 (0.47, 1.10) | 38 | 1.43 (0.49, 4.18) |

Abbreviation: ALND: axillary lymph node dissection; BCS: breast conserving surgery; ER+ oestrogen receptor positive; ET: endocrine therapy; sHR: subdistributional Hazard Ratio; iIBC: invasive ipsilateral breast cancer; iCBC: invasive contralateral breast cancer; RT: radiotherapy; SLNB: sentinel lymph node biopsy.

Supplementary Table 4. The multivariable Fine and Gray proportional subdistribution hazards analysis of association of factors with IBE and iIBC in women with DCIS treated with BCS+/-RT.

| **Characteristic** | **Categories** | **IBE** | | **iIBC** | |
| --- | --- | --- | --- | --- | --- |
|  |  | **Events** | **sHR (95% CI)** | **Events** | **sHR (95% CI)** |
| Age group | 45-69 years | 270 | Reference | 158 | Reference |
|  | <45 years | 35 | **1.80 (1.18, 2.75)** | 21 | **1.81 (1.08, 3.06)** |
|  | >69 years | 25 | 0.82 (0.52, 1.29) | 17 | 0.91 (0.53, 1.58) |
| Ethnicity | European | 231 | Reference | 145 | Reference |
|  | Māori | 35 | **1.54 (1.06, 2.23)** | 19 | 1.31 (0.79, 2.17) |
|  | Pacific | 20 | 1.42 (0.86, 2.33) | 10 | 1.13 (0.56, 2.25) |
|  | Asian | 42 | 1.01 (0.72, 1.42) | 21 | 0.83 (0.52, 1.32) |
|  | Other or Unknown | <5 | 0.46 (0.12, 1.77) | <5 | 0.40 (0.06, 2.75) |
| Deprivation | 1-4 (less deprived) | 156 | Reference | 90 | Reference |
|  | 5-7 | 94 | 1.07 (0.82, 1.40) | 54 | 1.09 (0.76, 1.55) |
|  | 8-10 (more deprived) | 80 | 1.07 (0.79, 1.45) | 52 | 1.32 (0.89, 1.96) |
| Rurality | Urban/other | 290 | Reference | 174 | Reference |
|  | Rural | 40 | 0.99 (0.70, 1.38) | 22 | 0.94 (0.61, 1.45) |
| Mode of diagnosis | Programming screen | 171 | Reference | 92 | Reference |
|  | non-Programming image | 95 | 0.85 (0.64, 1.13) | 65 | 0.96 (0.66, 1.39) |
|  | Symptomatic | 64 | 1.21 (0.85, 1.72) | 39 | 1.30 (0.83, 2.03) |
| DCIS size | ≤20mm | 240 | Reference | 148 | Reference |
|  | >20mm | 90 | **1.55 (1.19, 2.03)** | 48 | 1.33 (0.93, 1.89) |
| Grade | High | 117 | Reference | 65 | Reference |
|  | Intermediate | 136 | 1.01 (0.77, 1.33) | 83 | 1.10 (0.76, 1.59) |
|  | Low | 74 | 0.85 (0.57, 1.26) | 47 | 0.98 (0.59, 1.63) |
|  | Unknown | <5 | 0.47 (0.14, 1.59) | <5 | 0.25 (0.03, 1.94) |
| Necrosis | Present | 177 | Reference | 97 | Reference |
|  | None | 127 | 0.90 (0.67, 1.20) | 80 | 1.01 (0.69, 1.48) |
|  | Unknown | 26 | 0.74 (0.48, 1.15) | 19 | 0.95 (0.56, 1.63) |
| Locoregional treatment | BCS alone | 169 | Reference | 98 | Reference |
|  | BCS with RT | 161 | **0.56 (0.43, 0.72)** | 98 | **0.66 (0.47, 0.91)** |
| Facility of Surgery | Public | 182 | Reference | 110 | Reference |
|  | Private | 148 | **1.37 (1.06, 1.75)** | 75 | 1.35 (0.98, 1.87) |
|  | Unknown | 17 | 0.95 (0.55, 1.62) | 11 | 1.23 (0.61, 2.46) |
| Excision margin | ≥2mm | 266 | Reference | 161 | Reference |
|  | <2mm | 57 | 1.32 (0.97, 1.78) | 31 | 1.23 (0.82, 1.83) |
|  | Clear, unknown measurement | 7 | 1.11 (0.49, 2.52) | <5 | 0.88 (0.30, 2.59) |
| Axillary intervention | No axillary intervention | 283 | Reference | 164 | Reference |
|  | ALND | 5 | 1.85 (0.64, 5.36) | <5 | 1.86 (0.59, 5.84) |
|  | SLNB | 42 | 1.07 (0.75, 1.53) | 29 | 1.43 (0.92, 2.21) |
| ER status and adjuvant ET | ER+ without ET | 39 | Reference | 24 | Reference |
|  | ER+ with ET | 5 | 0.48 (0.18, 1.27) | <5 | 0.16 (0.02, 1.25) |
|  | Other | 286 | 0.95 (0.67, 1.35) | 171 | 0.95 (0.60, 1.49) |

Abbreviation: ALND: axillary lymph node dissection; BCS: breast conserving surgery; ER+: oestrogen receptor positive; ET: endocrine therapy; sHR: subdistributional Hazard Ratio; IBE: ipsilateral breast event; RT: radiotherapy; SLNB: sentinel lymph node biopsy.

Supplementary Table 5. The multivariable Fine and Gray proportional subdistribution hazards analysis of association of factors with any breast events in women with DCIS.

| **Characteristic** | **Categories** | **Events** | **sHR (95% CI)** |
| --- | --- | --- | --- |
| Age group | 45-69 years | 581 | Reference |
|  | <45 years | 83 | **1.66 (1.27, 2.16)** |
|  | >69 years | 49 | 0.82 (0.60, 1.14) |
| Ethnicity | European | 516 | Reference |
|  | Māori | 56 | 1.11 (0.84, 1.48) |
|  | Pacific | 37 | 1.21 (0.85, 1.72) |
|  | Asian | 94 | 1.01 (0.81, 1.26) |
|  | Other or Unknown | 10 | 0.93 (0.51, 1.70) |
| Deprivation | 1-4 (less deprived) | 350 | Reference |
|  | 5-7 | 206 | 1.05 (0.88, 1.26) |
|  | 8-10 (more deprived) | 157 | 0.98 (0.80, 1.20) |
| Rurality | Urban/other | 628 | Reference |
|  | Rural | 85 | 0.94 (0.74, 1.18) |
| Mode of diagnosis | Programming screen | 372 | Reference |
|  | non-Programming image | 205 | **0.81 (0.67, 0.99)** |
|  | Symptomatic | 136 | 0.87 (0.69, 1.11) |
| DCIS size | ≤20mm | 432 | Reference |
|  | >20mm | 281 | **1.30 (1.08, 1.57)** |
| Grade | High | 297 | Reference |
|  | Intermediate | 264 | 1.02 (0.85, 1.22) |
|  | Low | 145 | 0.97 (0.74, 1.28) |
|  | Unknown | 7 | 0.76 (0.35, 1.66) |
| Necrosis | Present | 411 | Reference |
|  | None | 250 | 0.98 (0.80, 1.21) |
|  | Unknown | 52 | **0.69 (0.51, 0.93)** |
| Locoregional treatment | BCS alone | 244 | Reference |
|  | BCS with RT | 273 | **0.70 (0.57, 0.85)** |
|  | Mastectomy | 196 | **0.43 (0.33, 0.56)** |
| Facility of Surgery | Public | 425 | Reference |
|  | Private | 257 | **1.32 (1.11, 1.57)** |
|  | Unknown | 31 | 0.83 (0.57, 1.21) |
| Excision margin | ≥2mm | 586 | Reference |
|  | <2mm | 113 | **1.34 (1.09, 1.65)** |
|  | Clear, unknown measurement | 14 | 1.05 (0.60, 1.85) |
| Axillary intervention | No axillary intervention | 487 | Reference |
|  | ALND | 21 | 0.97 (0.61, 1.57) |
|  | SLNB | 205 | 1.14 (0.92, 1.42) |
| ER status and adjuvant ET | ER+ without ET | 80 | Reference |
|  | ER+ with ET | 11 | **0.51 (0.27, 0.97)** |
|  | Other | 622 | 0.91 (0.72, 1.15) |

Abbreviation: ALND: axillary lymph node dissection; BCS: breast conserving surgery; ER+: oestrogen receptor positive; ET: endocrine therapy; sHR: subdistributional Hazard Ratio; IBE: ipsilateral breast event; RT: radiotherapy; SLNB: sentinel lymph node biopsy.
